# Effect of Cigarette Smoking and Passive Smoking on Hearing Impairment: Data from a Population–Based Study

**DOI:** 10.1371/journal.pone.0146608

**Published:** 2016-01-12

**Authors:** Jiwon Chang, Namhyung Ryou, Hyung Jin Jun, Soon Young Hwang, Jae-Jun Song, Sung Won Chae

**Affiliations:** 1 Department of Otolaryngology Head and Neck Surgery, Hallym University College of Medicine, Chuncheon, Korea; 2 Department of Otolaryngology-Head and Neck Surgery, Korea University College of Medicine, Seoul, Korea; 3 Department of Medical Statistics, Korea University College of Medicine, Seoul, Korea; University of Chicago, UNITED STATES

## Abstract

**Objectives:**

In the present study, we aimed to determine the effect of both active and passive smoking on the prevalence of the hearing impairment and the hearing thresholds in different age groups through the analysis of data collected from the Korea National Health and Nutrition Examination Survey (KNHANES).

**Study Design:**

Cross-sectional epidemiological study.

**Methods:**

The KNHANES is an ongoing population study that started in 1998. We included a total of 12,935 participants aged ≥19 years in the KNHANES, from 2010 to 2012, in the present study. Pure-tone audiometric (PTA) testing was conducted and the frequencies tested were 0.5, 1, 2, 3, 4, and 6 kHz. Smoking status was categorized into three groups; current smoking group, passive smoking group and non-smoking group.

**Results:**

In the current smoking group, the prevalence of speech-frequency bilateral hearing impairment was increased in ages of 40−69, and the rate of high frequency bilateral hearing impairment was elevated in ages of 30−79. When we investigated the impact of smoking on hearing thresholds, we found that the current smoking group had significantly increased hearing thresholds compared to the passive smoking group and non-smoking groups, across all ages in both speech-relevant and high frequencies. The passive smoking group did not have an elevated prevalence of either speech-frequency bilateral hearing impairment or high frequency bilateral hearing impairment, except in ages of 40s. However, the passive smoking group had higher hearing thresholds than the non-smoking group in the 30s and 40s age groups.

**Conclusion:**

Current smoking was associated with hearing impairment in both speech-relevant frequency and high frequency across all ages. However, except in the ages of 40s, passive smoking was not related to hearing impairment in either speech-relevant or high frequencies.

## Introduction

Hearing loss is one of the most common sensory impairments, and results from pathological conditions along the auditory pathway [1]. Hearing impairment hampers the ability to understand speech, and leads to difficulties in communication and social connectivity. The prevalence of hearing impairment is increasing and the World Health Organization (WHO) reported that 360 million people, which exceeds is over 5% of the worlds’ population, have disabling hearing loss (defined as an average hearing threshold of ≥ 40dBHL), and that one-third of people over 65 years old are affected by disabling hearing loss [2]. In our previous study, the prevalence of hearing loss (defined as an average hearing threshold of ≥ 25dBHL) in speech-relevant frequencies (0.5, 1, 2, and 4kHz) was 9.31% for unilateral hearing loss and 13.42% for bilateral hearing loss [3].

There are various risk factors for hearing loss; genetic causes, complications at birth, infectious disease, chronic ear infections, the use of ototoxic medications, exposure to noise, sex, ageing and so on. Among the different associated factors, ageing is a well-known and major factor in hearing loss [4, 5]. Age-related hearing loss usually begins in the third decade of life, progresses gradually, and typically involves the hearing threshold at high frequencies. Additionally, men are reported to have a higher risk for developing hearing loss [3, 5–7] probably due to their greater likelihood of exposure to extrinsic ototoxic insults. On the other hand, while smoking is a well-known risk factor for many health problems, the association of cigarette smoking and hearing loss has been inconsistent [8–13]. A previous study has shown that smoking is correlated with hearing loss in the geriatric population [8]. Other studies found that smoking pack-years and ageing have multiplicative effects on developing hearing impairment [9]. However, older people have mostly smoked cigarettes for a longer period than younger people, and therefore a long duration of smoking would have affected the cochlear circulation more and could thereby result in a high prevalence of hearing loss. From this perspective, it is essential to evaluate the hearing loss among smokers categorized by age and with adjustment for the age.

In this present study, we aimed to determine the effect of smoking on the prevalence of hearing impairment in different age groups among the general population. We also investigated the influence of both current and passive smoking on the prevalence of hearing impairment and hearing thresholds.

## Methods

### Study Population

The Korea National Health and Nutrition Examination Survey (KNHANES) commenced in 1998, and collates the general health and nutrition status of populations under the auspices of the Korean Ministry of Health and Welfare. KNHANES V is the fifth survey and represents data for the years 2010 to 2012. KNHANES V used a rolling sample design, so that the samples from each year were independent and represented the whole South Korean population. KNHANES V included 11,520 South Korean households. In the present study, a total of 12,935 individuals aged 19 years and above, representing a South Korean population of 27,435,476, were included.

### Audiometric Measurement and the Definition of Hearing Impairment

Pure-tone audiometric tests were conducted using a SA 203 audiometer (Entomed; Malmö, Sweden). Tests were performed in a soundproof booth inside a mobile bus reserved for the KNHANES, using supra-auricular headphones. Otolaryngologists, who had been trained to operate the audiometer, provided instructions to participants and obtained the recordings. Automated testing was programmed according to a modified Hughson−Westlake procedure; it used a single pure tone of 1 to 2 seconds and the lowest level at which the subject responded to 50% of the pure tone was set as the threshold. The automated hearing test involving air-conducted pure tone stimuli showed good test–retest reliability and validity, comparable to the manual pure-tone audio test [14].Participants reacted by pushing a button when they heard a tone and the results were automatically recorded. The frequency ranges tested were 0.5, 1, 2, 3, 4, and 6 kHz.

We assessed two types of hearing impairment for the analysis; speech-relevant frequency hearing impairment and high-frequency hearing impairment. Speech-relevant frequency hearing impairment was defined as a threshold of ≥25 decibel hearing level (dBHL) in pure tone audiometry (PTA) of frequencies at 0.5, 1, 2, 3, and 4 kHz. High-frequency hearing impairment often precedes speech-relevant hearing impairment since it is more susceptible to environmental ototoxic factors, and was defined as a threshold of ≥ 25 dBHL in PTA of frequencies at 3, 4, and 6 kHz.

In the study, only bilateral hearing impairment cases were included; and the impairment was defined as a hearing threshold ≥25 dBHL in both ears. To minimize the influence of age on hearing level, we categorized the participants into similar age groups and evaluated the prevalence of hearing impairment and the decline in hearing thresholds.

### Definition of Smoking

Smoking status was categorized into three groups. The current smoking group was defined as participants who smoke at present time regardless of the amount they inhale. The passive smoking group was described as participants who have never smoked in their lifetime, but were exposed to daily cigarette fumes daily at home and/or at work, regardless of the exposure time. The non-smoking group was characterized as individuals who had never smoked in their lifetime and were was not exposed to cigarette smoke at home or work.

### Lifestyle Habits

Medical history information and lifestyle habits were collected using physical examinations and self-report questionnaires. The experience of the work-related noise exposure was asked regardless of the exposure time. The prevalence of diabetes, hypertension, depression, and stress were identified. Regular exercise was defined as moderate physical activity performed for at least 30 minutes at a time at least five times a week.

### Statistical Analysis

We estimated the prevalence of bilateral hearing impairment in both speech-relevant frequency and high-frequency according to the smoking status. Analysis was conducted under stratification by age group (19−29, 30−39, 40−49, 50−59, 60−69, 70−79, ≥ 80 years). We also calculated the PTA thresholds in both speech-relevant frequency and high-frequency according to the smoking status. The differences between groups were analyzed using the χ2 (chi-square) method and linear regression analysis with adjustment for age, sex, and work-related noise exposure. Logistic regression analysis was performed to determine the odds ratio (OR) and 95% confidence interval (CI), with adjustment for sex, age, and work-related noise exposure. We first adjusted for age and sex (model 1), and then adjusted for age, sex, and work-related noise exposure (model 2), and finally adjusted for age, sex, work-related noise exposure, diabete, hypertension, depression, stress and regular exercise (model 3). Statistical analyses were performed using the survey procedure instructions for the SAS 9.3 software (SAS Institute; Cary, NC, USA). P-values < 0.05 were considered statistically significant. All data were analyzed using the survey sample weights assigned to represent the Korean population.,

## Results

### Characteristics of the study population

Among 12,935 participants analyzed, 3,374 (26.08%) were current smokers, 2,792 (21.58%) were passive smokers and 6,769 (52.33%) were non-smokers (Table 1). Of the participants, 1,683 (13.01%) were aged between 19 and 29, 2,570 (19.87%) were in their 30s, 2,401 (18.56%) were in their 40s, 2,550 (19.91%) were in their 50s, 2,041 (15.78%) were in their 60s, 1,445 (11.17%) were in their 70s and 245 (1.89%) were over 80 years old.

**Table 1:** Demographic characteristics of participants in the Korea National Health and Nutrition Examination Survey (KNHANES).

Age group, yr	Current Smoking	Passive smoking	Non-smoking	Total (Unweighted frequency of participants)	(%)	Total (weighted frequency of participants)
19–29	474	504	705	1,683	13.01	5,810,127
30–39	860	568	1,142	2,570	19.87	5,987,461
40–49	697	614	1,090	2,401	18.56	6,027,489
50–59	628	619	1,303	2,550	19.91	4,872,522
60–69	407	326	1,308	2,041	15.78	2,627,753
70–79	272	148	1,025	1,445	11.17	1,762,308
80-	36	13	196	245	1.89	347,817
Total	3,374	2,792	6,769	12,935	100	27,435,476

%: percent of total (unweighted frequency of participants)

### Smoking is related to speech frequency bilateral hearing impairment in the 40s, 50s, and 60s age- groups

**Fig 1:**
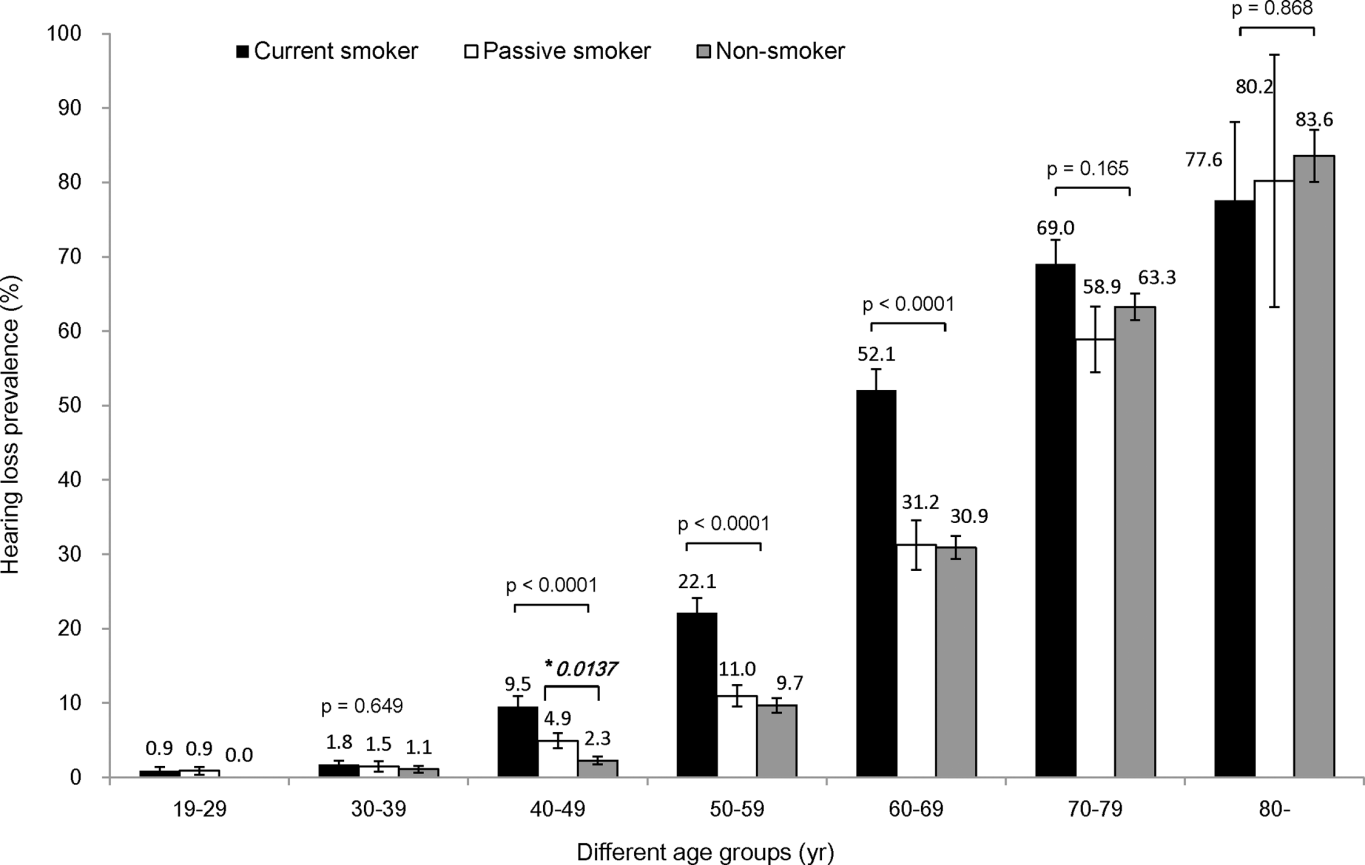
The prevalence of speech relevant frequency bilateral hearing impairment according to smoking status. Cigarette smoking was associated with speech-relevant frequency hearing impairment in the 40s, 50s, and 60s age-groups. The current smoking group had a higher prevalence of hearing impairment than the passive smoking and non-smoking groups for these age groups. In the 40s age-group, the prevalence of hearing impairment was 9.5% in the current smoking group, 4.9% in the passive smoking group and 2.3% in the non-smoking group. In the 50s age-group, the prevalence of hearing impairment was 22.1% in the current smoking group, 11.0% in the passive smoking group and 9.7% in the non-smoking group. In the 60s age-group, the prevalence of hearing impairment was 52.1% in the current smoking group, 31.2% in the passive smoking group and 30.9% in the non-smoking group. However, in the 19−29, 30s, 70s, and ≥ 80 years age-groups, there were no statistically significant differences in the prevalence of hearing impairment among the three smoking status categories. Passive smoking did not significantly increase the prevalence of hearing impairment, except in the 40s age-group. In this group, passive smoking (4.9%) elevated the prevalence of hearing impairment, as compared to non-smoking (2.3%: *p = 0.0137).

In age-stratified analysis, cigarette smoking was associated with speech-relevant frequency hearing impairment in 40s, 50s, and 60s age-group (Fig 1). The current smoking group had a higher prevalence of hearing impairment than the passive smoking and non- smoking groups in these age-groups. Passive smoking did not significantly increase the prevalence of hearing impairment, except for the 40s age-group.

After adjustment for age, and sex, the OR of smoking status was evaluated. The OR of the current smoking group was 1.48, with a 95% CI of 1.16 − 1.88 (Table 2, Model 1). After adjustment for age, sex and work-related noise exposure, the OR of the current smoking group was 1.44, with a 95% CI of 1.13 − 1.83 (Model 2). After adjustment for age, sex, work-related noise exposure, diabete, hypertension, depression, stress and regular exercise, the OR of the current smoking group was 1.39, with a 95% CI of 1.08 − 1.79 (Model 3). Thus, the prevalence of speech-frequency bilateral hearing impairment was significantly higher in the current smoking group than in the non- smoking group. However, the prevalence of hearing impairment in the passive smoking group was higher than that in the non- smoking group, but the difference was not statistically significant (Model 1 OR 1.08, 95% CI 0.88 − 1.33; Model 2 OR 1.06, 95% CI 0.86 − 1.30; Model 3 OR 1.09, 95% CI 0.88 − 1.35).

**Table 2:** Adjusted odds ratio for prevalence of hearing impairment ^¶^ according to smoking status.

Types of hearing loss	Smoking Status	Model 1			Model 2			Model 3		
		OR	95% CI	p value	OR	95% CI	p value	OR	95% CI	p value
Bilateral hearing loss at speech frequencies	Current smoker	1.48	1.16–1.88	0.0016	1.44	1.13–1.83	0.0033	1.39	1.08–1.79	0.0098
	Passive smoker	1.08	0.88–1.33	0.4595	1.06	0.86–1.30	0.5944	1.09	0.88–1.35	0.4095
	Non-smoker	1			1					
Bilateral hearing loss at high frequencies	Current smoker	1.41	1.13–1.74	0.0019	1.38	1.11–1.71	0.0032	1.42	1.13–1.77	0.0024
	Passive smoker	1.08	0.91–1.30	0.381	1.07	0.89–1.28	0.4673	1.12	0.93–1.35	0.2151
	Non-smoker	1			1					

Abbreviations: CI confidence interval; OR: odds ratio

^**¶**^ Adjusted for age, sex, and work-related noise exposure

Model 1: Adjusted for age, and sex

Model 2: Adjusted for age, sex, and work-related noise exposure

Model 3; Adjusted for age, sex, work-related noise exposure, diabete, hypertension, depression, stress, and moderate physical activity.

### Smoking is related to high frequency bilateral hearing impairment in the 30s, 40s, 50s, 60s, and 70s age-Groups

**Fig 2:**
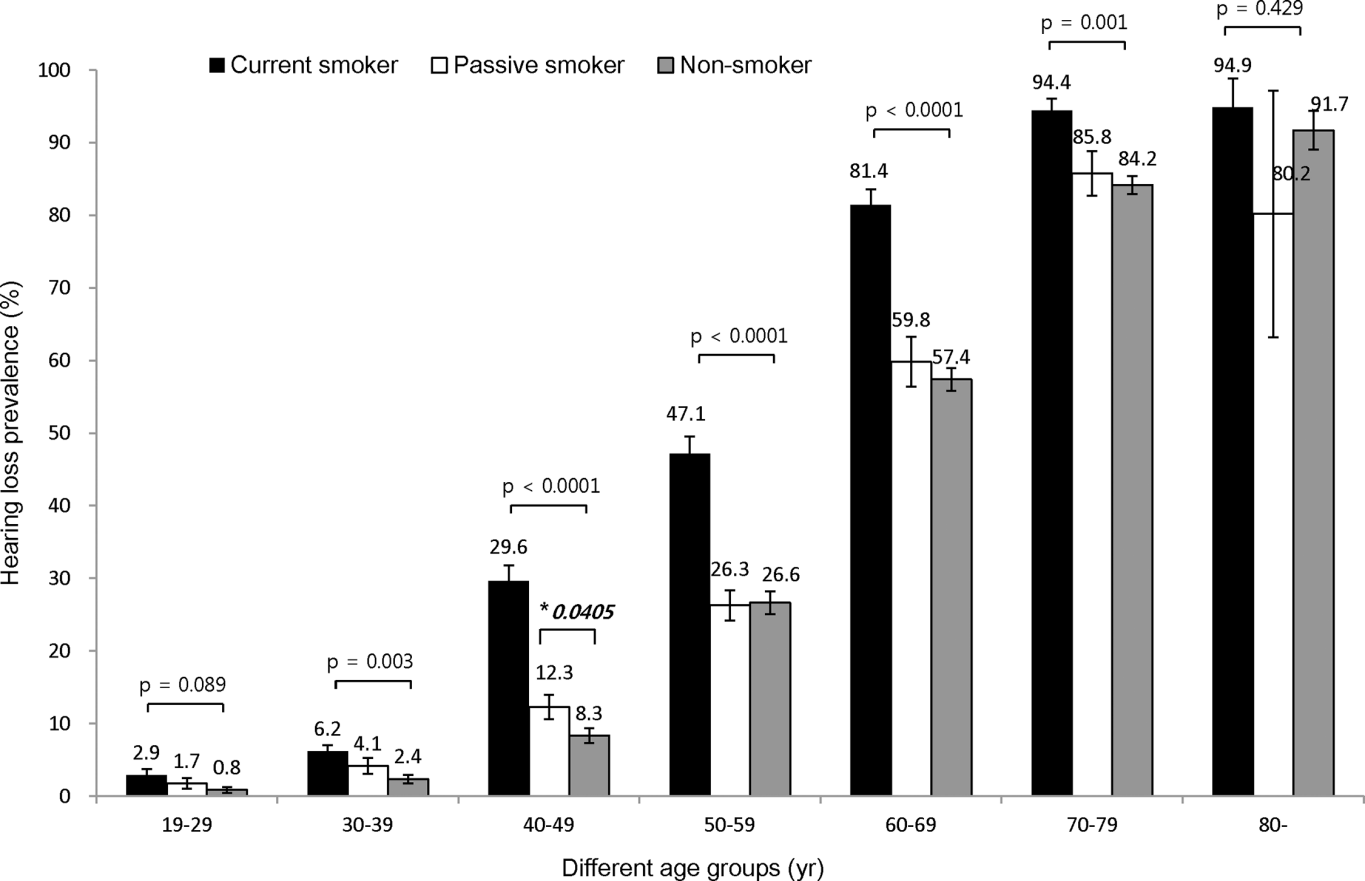
The prevalence of high frequency bilateral hearing impairment according to smoking status. Smoking showed an association with high frequency bilateral hearing impairment in the 30s, 40s, 50s, 60s, and 70s age-groups. The current smoking group had a higher prevalence of hearing impairment than the passive smoking group and non-smoking group in these age-groups. In the 30s age-group, the prevalence of hearing impairment was 6.2% in the current smoking group, 4.1% in the passive smoking group and 2.4% in the non-smoking group. In the 40s age-group, the prevalence of hearing impairment was 29.6% in the current smoking group, 12.3% in the passive smoking group and 8.3% in the non-smoking group. In the 50s age-group, the prevalence of hearing impairment was 47.1% in the current smoking group, 26.3% in the passive smoking group and 26.6% in the non- smoking group. In the 60s age-group, the prevalence of hearing impairment was 81.4% in the current smoking group, 59.8% in the passive smoking group and 57.4% in the non- smoking group. In the 70s age-group, the prevalence of hearing impairment was 94.4% in the current smoking group, 85.8% in the passive smoking group and 84.2% in the non- smoking group. However, in the 19−29 years and ≥ 80 years age-groups, there was no statistically significant differences in the hearing impairment prevalence among the three smoking status categories. Passive smoking elevated the prevalence of high frequency hearing impairment in the 40s age-group (*p = 0.0405). In this group, passive smoking (12.3%) elevated the prevalence of hearing loss compared to non-smoking (8.3%) (p = 0.0405). However, passive smoking did not increase the prevalence of high frequency hearing loss in other age groups.

In age-stratified analysis, cigarette smoking demonstrated a close relationship with the prevalence rate of high frequency hearing loss in the 30s, 40s, 50s, 60s, and 70s age groups (Fig 2). The current smoking group had a higher prevalence of hearing impairment than the passive smoking and non- smoking group in these age groups. Passive smoking did not significantly increase the prevalence of high frequency hearing impairment except in the 40s age-group.

After adjustment for age, and sex, the OR of smoking status was evaluated. The OR of the current smoking group was 1.41, with a 95% CI of 1.13 − 1.74 (Table 2, Model 1). After adjustment for age, sex and work-related noise exposure, the OR of current smoking group was 1.38, with a 95% CI of 1.11 − 1.71 (Model 2). After adjustment for age, sex, work-related noise exposure, diabete, hypertension, depression, stress and regular exercise, the OR of the current smoking group was 1.42, with a 95% CI of 1.13 − 1.77 (Model 3). Thus, the prevalence of high frequency bilateral hearing impairment was significantly higher in the current smoking group than in the non- smoking group. The prevalence of hearing impairment in the passive smoking group was higher than that in the non- smoking group, but the this difference was not significant (Model 1 OR 1.08, 95% CI 0.91 − 1.30; Model 2 OR 1.07, 95% CI 0.89 − 1.28; Model 3 OR 1.12, 95% CI 0.93 − 1.35).

### Smoking exposure influenced hearing thresholds

In order to investigate the impact of smoking on hearing thresholds, we compared the PTA thresholds of individuals in the three smoking categories.

**Fig 3:**
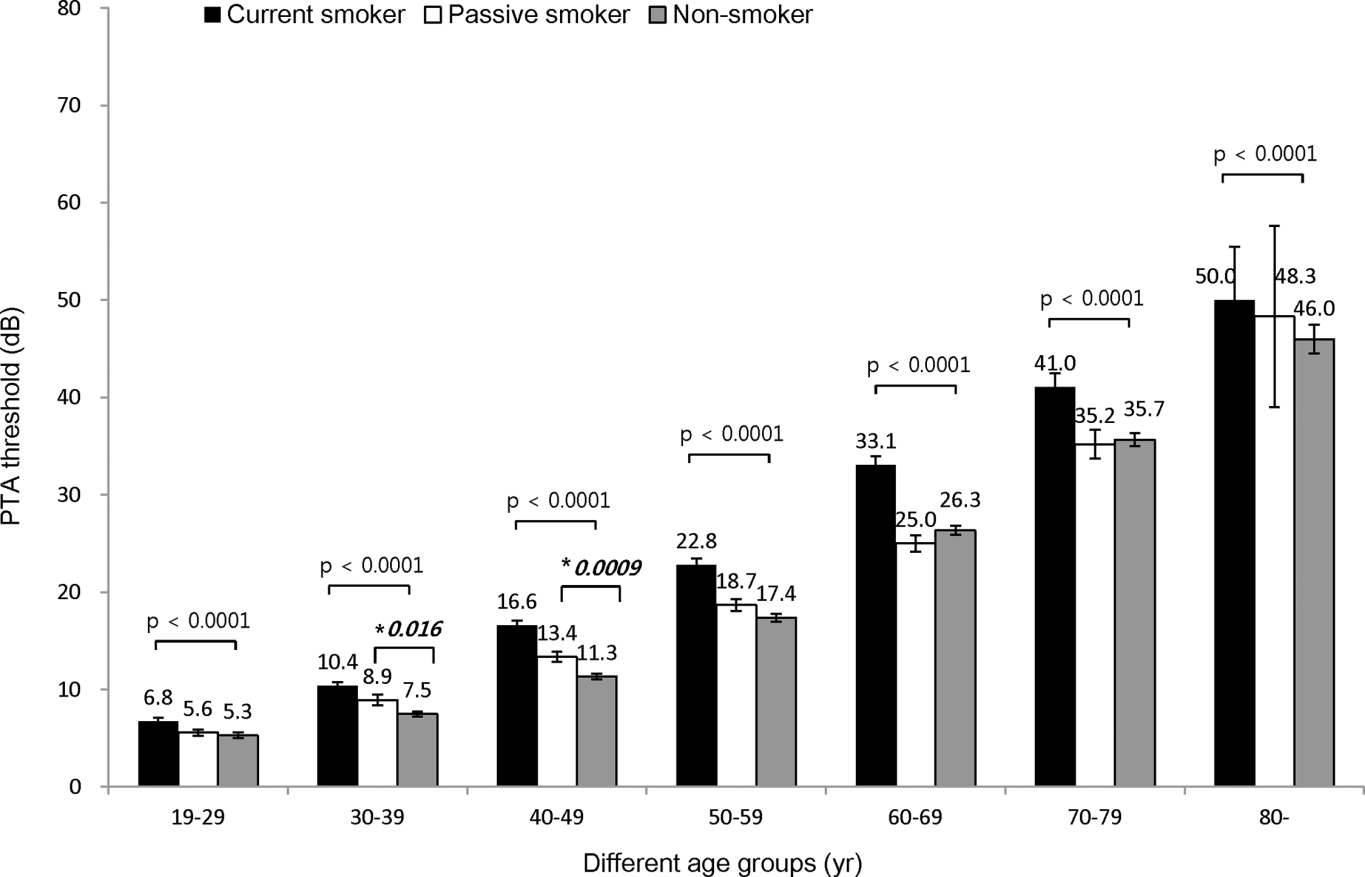
Effect of smoking on PTA thresholds at speech-relevant frequencies. The current smoking group had significantly increased hearing threshold than the passive smoking group and the non- smoking group throughout all ages. The passive smoking group had higher hearing thresholds than the non-smoking group in 30s (8.9dB in passive smoking vs 7.5dB in non-smoking) (*p = 0.016) and 40s (13.4dB in passive smoking vs. 11.3dB in non-smoking) (*p = 0.0009) age groups and it was statistically significant.

**Fig 4:**
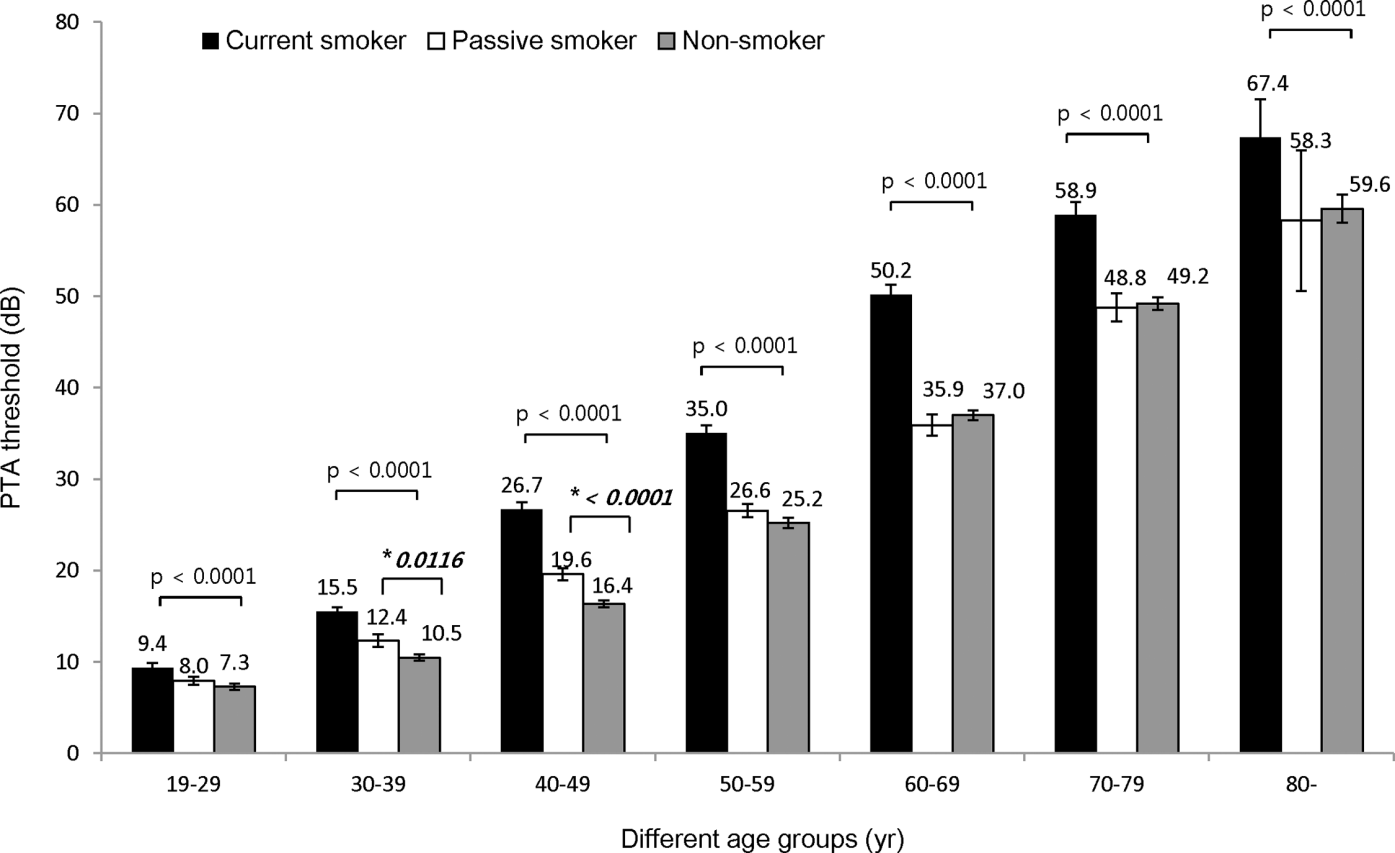
Effect of smoking on PTA threshold at high frequencies. In the analysis of hearing threshold at high frequencies, hearing thresholds were elevated in the current smoking group compared to those in the passive smoking group and non- smoking group, across all ages. Additionally, the passive smoking group had significantly higher hearing thresholds than the non- smoking group in the 30s (12.4dB in the passive smoking vs 10.5dB in the non-smoking groups; *p = 0.0116) and the 40s (19.6dB in the passive smoking vs 16.4dB in the non-smoking groups; *p < 0.0001) age-groups.

Fig 3 demonstrates the differences in the PTA thresholds at speech-relevant frequencies, and Fig 4 shows the differences of PTA thresholds at the high frequency hearing thresholds.

The association of smoking with hearing threshold was further examined with regression analysis. In the analysis of speech-relevant frequencies after adjusting for age, sex, and work-related noise exposure, the current smoking group had a 2.46 ± 0.33 dB higher PTA threshold than the non- smoking group, and a 1.64 ± 0.34 dB higher threshold than the passive smoking group (Table 3). Moreover, the passive smoking group had a 0.81 ± 0.27 dB higher PTA threshold than the non- smoking group, which was statistically significant (p = 0.0026). Similar results were obtained in the analysis of the hearing thresholds at high frequencies. The current smoking group had higher PTA thresholds than the non- smoking group (3.60 ± 0.43 dB) and the passive smoking group (2.41 ± 0.47 dB); and the passive smoking group had higher PTA thresholds than the non- smoking group (1.18 ± 0.34 dB) which was statistically significant (p = 0.0007).

**Table 3:** Adjusted^¶^ effect of smoking on hearing thresholds.

Range of hearing frequencies	Smoking Status	Categories Compared	Mean Difference in Hearing threshold ± SE	p value
Speech relevant frequencies	Current smoker	Non-smoker	2.46 ± 0.33	< 0.0001
	Current smoker	Passive smoker	1.64 ± 0.34	< 0.0001
	Passive smoker	Non-smoker	0.81 ± 0.27	0.0026
High frequencies	Current smoker	Non-smoker	3.60 ± 0.43	< 0.0001
	Current smoker	Passive smoker	2.41 ± 0.47	< 0.0001
	Passive smoker	Non-smoker	1.18 ± 0.34	0.0007

Abbreviation: SE; Standard error

^**¶**^ Adjusted for age sex and work-related noise exposure

## Discussion

To our knowledge, this is the first study demonstrating the age-specific effect of both active and passive smoking on the prevalence of hearing impairment and hearing thresholds in a large population survey study. In this study, current smoking was found to increase the prevalence of speech-relevant frequency hearing impairment in individuals aged 40−69 years, and the rate of high frequency hearing impairment in individuals aged 30−79 years. Additionally, current smoking significantly affected hearing thresholds across all ages in both speech-relevant and high frequencies. On the other hand, passive smoking had a significant effect only on the prevalence of hearing impairment in individuals aged 40−49 years, and elevated the hearing threshold of speech-relevant frequencies and high frequencies in the 30s and 40s age groups. Moreover, regression analysis of the association between smoking and hearing threshold demonstrated that both the current smoking group and passive smoking group had higher hearing thresholds than the non-smoking group.

Hearing impairment is affected by various social factors, such as smoking, alcohol use, occupational or leisure noise exposure, and occupational vibration, as well as different medical factors, such as hypertension, diabetes, and increased serum cholesterols [5, 15–18]. It is obvious from our data and other studies [4, 5] that the incidence of hearing impairment increases with age regardless of smoking habits. However, we identified that, in the same age group, current smoking significantly increases the rate of hearing impairment. In the present analysis, the prevalence of speech-relevant frequency hearing impairment in the current smoking group was higher in individuals in their 40s, 50s, and 60s.

We suggest that the reason that this rate was not increased in younger age groups is that the duration of smoking was not sufficiently long to develop speech-frequency hearing impairment. Additionally, the reason that current smoking did not affect the prevalence of speech-frequency hearing impairment in older groups may be related to the reduced number of participants, the effects of selective mortality and the influences of multiplicative factors in these age groups. These suggestions are supported by the fact that the influence of current smoking is greater in individuals in their 50s and 60s than those in their 40s.

High frequency hearing impairment preceded the speech-relevant hearing impairment and the current smoking group showed increased prevalence of high frequency hearing impairment starting with individuals in the 30s age-groups and older. These results are consistent with other studies reporting that cigarette affects hearing levels more prominently at high frequencies [19–21]. Another study reported that cigarette smoking had raised the hearing threshold in young adults aged between 21–23 years, in terms of higher frequencies in the audiometry [20]. A specialized high frequency audiometric test over 6 kHz was not used in our study, since our survey was conducted national wide targeting the general population, and these methodological differences may account for the discrepant results among the studies.

Some studies have reported correlations between passive smoking and hearing loss prenatally, and in infants, children, and adolescents [22–24]. Exposure to environmental tobacco in childhood is suggested to affect the cochlear physiology by affecting the outer hair cells which can be measured through a decreased response in transient evoked otoacoustic emissions (TEOAE) [22]. The reported PTA threshold measured after exposure to tobacco is around 20−25 dBHL, indicating that passive smoking is related to mild or minimal sensorineural hearing loss [23]. There are few available studies conducted in adults, but these report that passive exposure to tobacco is more likely to result in hearing loss [15, 25]. However, these studies defined hearing loss with speech-in-noise test or included former smokers into the category of passive smokers.

According to our analysis, individuals in the passive smoking group (defined as participants who are exposed to cigarette smoke at home and at work, and who had never personally smoked before) had significantly increased hearing thresholds compared to those in the non-smoking group, but passive smoking only increased the prevalence of both speech-relevant frequency and high frequency hearing impairment (hearing thresholds higher than 25dBHL) in the age group of 40−49 years. The reason for the affectation of this particular age group is unclear, but since this age group is considered to be the most socially active in Korea, it is possible that such individuals have a high chance of being exposed to large amounts of cigarette smoke in the working place. However, this result could also be biased as this analysis was based on data obtained from self-reported questionnaires regarding passive smoking, and it was not possible to quantitate the extent of the exposure.

A major advantage of our study is that this is an analysis of a large, population-based, nationally representative samples of adults. To the best of our knowledge, there are few other studies reporting a national-level evaluation of the relationship of smoking and passive smoking with hearing loss using audiometric testing. There are some limitations to the present study. First, since it is a cross-sectional study, we cannot identify the causal relationship or the time-course of smoking and the development of hearing loss. Second, our study used KNHANES data, which uses self-reported questionnaires to examine the respondents’ lifestyle, which can create a recall bias. Third, since the amount and the duration of smoking exposure were not considered, we could not infer the quantitative relationship. Finally, we defined high frequency hearing loss as a hearing loss at 3, 4, and 6 kHz. Extended high-frequency audiometry above 8 kHz would provide clearer information regarding hearing loss at higher frequencies.

## Conclusion

Current smoking was associated with hearing impairment in both speech-relevant frequency and high frequency across all ages. However, except for individuals in their 40s, passive smoking was not related to hearing impairment in either speech-relevant or high frequencies.
